# Altered muscle activation patterns (AMAP): an analytical tool to compare muscle activity patterns of hemiparetic gait with a normative profile

**DOI:** 10.1186/s12984-019-0487-y

**Published:** 2019-01-31

**Authors:** Shraddha Srivastava, Carolynn Patten, Steven A. Kautz

**Affiliations:** 1 Department of Health Sciences and Research, College of Health Professions, Medical University of South Carolina, 77 President Street, Charleston, SC 29425 USA; 2 Ralph H. Johnson VA Medical Center, Charleston, SC 29401 USA; 3 Biomechanics, Rehabilitation, and Integrative Neuroscience (BRaIN) Lab, Department of Physical Medicine and Rehabilitation, University of California Davis School of Medicine, Sacramento, CA 95817 USA; 4 VA Northern California Health Care System, Martinez, CA 94553 USA

**Keywords:** Stroke, Locomotion, Electromyography, EMG assessment, Muscle activation patterns

## Abstract

**Background:**

Stroke survivors often have lower extremity sensorimotor impairments, resulting in an inability to sufficiently recruit muscle activity at appropriate times in a gait cycle. Currently there is a lack of a standardized method that allows comparison of muscle activation in hemiparetic gait post-stroke to a normative profile.

**Methods:**

We developed a new tool to quantify altered muscle activation patterns (AMAP). AMAP accounts for spatiotemporal asymmetries in stroke gait by evaluating the deviations of muscle activation specific to each gait sub-phase. It also recognizes the characteristic variability within the healthy population. The inter-individual variability of normal electromyography (EMG) patterns within some sub-phases of the gait cycle is larger compared to others, therefore AMAP penalizes more for deviations in a gait sub-phase with a constant profile (absolute active or inactive) vs variable profile. EMG data were collected during treadmill walking, from eight leg muscles of 34 stroke survivors at self-selected speeds and 20 healthy controls at four different speeds. Stroke survivors’ AMAP scores, for timing and amplitude variations, were computed in comparison to healthy controls walking at speeds matched to the stroke survivors’ self-selected speeds.

**Results:**

Altered EMG patterns in the stroke population quantified using AMAP agree with the previously reported EMG alterations in stroke gait that were identified using qualitative methods. We defined scores ranging between ±2.57 as “normal”. Only 9% of healthy controls were outside “normal” window for timing and amplitude. Percentages of stroke subjects outside the “normal” window for each muscle were, Soleus = 79%; 73%, Medial Gastrocnemius = 62%; 79%, Tibialis Anterior = 62%; 59%, and Gluteus Medius = 48%; 51% for amplitude and timing component respectively, alterations were relatively smaller for the other four muscles. Paretic-propulsion was negatively correlated to AMAP scores for the timing component of Soleus. Stroke survivors’ self-selected walking speed was negatively correlated with AMAP scores for amplitude and timing of Soleus but only amplitude of Medial gastrocnemius (*p* < 0.05).

**Conclusions:**

Our results validate the ability of AMAP to identify alterations in the EMG patterns within the stroke population and its potential to be used to identify the gait phases that may require more attention when developing an optimal gait training paradigm.

**Trial registration:**

ClinicalTrials.gov NCT00712179, Registered July 3rd 2008

**Electronic supplementary material:**

The online version of this article (10.1186/s12984-019-0487-y) contains supplementary material, which is available to authorized users.

## Background

While a comprehensive, quantitative understanding of walking in the post-stroke population includes assessment of muscle activity patterns in addition to biomechanical variables and clinical measures, existing techniques to identify abnormal muscle activity have significant limitations. Current techniques do not account for altered gait mechanics (e.g., gait asymmetries that cause the same gait phase to occur at different percentages of the gait cycle between different hemiparetic subjects, as well as paretic, non-paretic or control legs); robustly identify the onset or termination of electromyographic (EMG) activity [1, 2]; or acknowledge normal gait variability [3]. So far, only a few tools have been proposed for quantitatively identifying EMG abnormalities during walking post-stroke [1, 2, 4, 5]. However, one significant limitation of all these tools is that they do not account for the asymmetries i.e. phase-specific differences between healthy and post-stroke walking patterns (we have further discussed the methodology and limitations of these tools in comparison to the proposed tool in the discussion section under *AMAP and existing tools for quantitative assessment of muscle activity*). Since phase-specific biomechanical demands constrain EMG activity, a methodology to evaluate EMG abnormalities must account for how prolonged or shortened phases of the gait cycle contribute to asymmetric gait post-stroke. A second limitation of existing techniques is that they typically lack a robust method for identifying EMG “On/Off” activity, and therefore can have poor inter-rater or intra-rater reliability leading to inaccuracies in detecting gait events in hemiparetic walking. A third limitation is the large inter-individual variability within the healthy population that is not accounted for by current methods, which may result in over estimation of EMG pattern abnormalities while evaluating deviations from an averaged normal profile. Here we seek to develop a quantitative measure that can recognize the characteristic variability within the healthy population while quantifying deviations in the amplitude and timing of stroke survivors’ EMG profiles accounting for asymmetric hemiparetic gait.

Impaired muscle activity associated with abnormal gait patterns is typical in stroke survivors. Abnormal muscle coordination post-stroke includes: temporal deviations in muscle activity patterns, for example, early plantar flexor activity during stance, or early termination of tibialis anterior activity during swing [6, 7], inappropriate level of activation due to insufficient muscle activity and weakness [8, 9], or co-activation of multiple muscles [6, 10]. Altered muscle activation patterns following stroke contribute to reduced gait speed [8, 11] and/or poor postural stability [12]. Therefore, understanding abnormalities of muscle coordination is important as dyscoordination can result in significant gait deficits that further limit functional walking capacity post-stroke.

Current evaluations of abnormalities in locomotor coordination tend to rely primarily upon biomechanical measures as opposed to quantitative measures of EMG activity. Biomechanical measures, while informative, do not provide specific insight into the altered muscle coordination patterns following stroke. For example, paretic propulsion is associated with functional walking ability [13]. Stroke survivors with lower levels of gait impairment demonstrate that SO, MG, and GM on the paretic side contribute towards forward propulsion which is similar to healthy individuals. But, stroke survivors with greater gait impairments and decreased muscle activity have limited contribution from SO, MG, and GM on the paretic side, and compensatory activity from RF and VM on the non-paretic side contributes towards forward propulsion [14]. Therefore, evaluating muscle activation patterns is important for clinicians to develop interventions that can target specific biomechanical task improvement. Recent studies have proposed analyses of modular organization of muscle activity [15, 16] that identifies distinct muscle groupings potentially corresponding to biomechanical functions [17, 18]. The modular organization has been used as a means to express motor coordination complexity during walking as a single integer value that represents the number of independently activated muscle groupings. However, a motor coordination complexity variable does not provide detailed information related to the abnormalities of individual muscle activity during walking. Furthermore, by limiting evaluation to a single integer value for each individual affords limited ability to differentiate between individuals. Therefore, there is a lack of a standard gait metric that can be used for a muscle-by-muscle evaluation to quantify the abnormalities of muscle coordination in hemiparetic gait.

An analytical tool that quantifies muscle patterns in hemiparetic gait needs to account for spatiotemporal gait asymmetries. Simulation studies of human walking based on computer-implemented musculoskeletal models have demonstrated that muscle activity in healthy individuals produce specific biomechanical outputs during the gait cycle [17, 19]. The biomechanical consequences of muscle activity during walking depend on the mechanical state of the leg. Although the mechanical state of the leg may have some inter-subject variation in a given phase due to the large variability within the stroke population, this variability is much less than the differences in activity between different phases of the gait cycle. Specifically, activation of a specific muscle during a given phase in post-stroke gait is likely to have more similar biomechanical consequences to activity in that same phase in a neurologically healthy person than to activity matched to a given percent of gait cycle (e.g., activity at the beginning of a swing phase occurring at 75% of the gait cycle in a person with hemiparesis is most accurately compared to activity in a neurologically healthy person at the beginning of swing phase occurring at ~ 60% as opposed to matched mid-swing activity occurring at 75% of time normalized gait cycle). However, existing methods for assessing EMG patterns for experimental walking data typically time normalize the data by expressing it as a percentage of the gait cycle [1, 2, 4]. While the alterations in muscle activity have a causal relationship with the biomechanical deviations, we posit that one cannot correctly evaluate deviations of EMG patterns without controlling for the deviations in the biomechanical consequences of muscle activity. Therefore, evaluation of muscle coordination should be specific to each gait sub-phase.

Typically used methods to identify periods of muscle activity/inactivity during normal walking have especially poor reliability within the stroke population. When identifying temporal deviations of the EMG patterns, the robustness of determining the “on/off” windows of muscle activity can greatly influence the outcome. One frequently proposed method to detect these windows of muscle activity is visual inspection which has been reported to have poor intra-rater reliability [20]. Additionally, some studies rely upon automated computer-based algorithms that use a pre-determined threshold, set using arbitrary criteria [21], which may not be robust enough to identify “on/off” events for many individuals. EMG activity has large variability across individuals, especially within the stroke population [6, 9, 22], therefore identifying a robust criterion to determine a single threshold value that can be reliable for identifying EMG onset or termination across subjects can be difficult. Recognizing the need for a method sufficiently sensitive to identify when a muscle is “on” or “off” is critical when developing an assessment tool for muscle timing abnormalities, especially in the stroke population.

Some sub-phases of the gait cycle reveal high inter-individual variability of EMG patterns relative to sub-phases with relatively consistent activity across healthy individuals, thus these phase-specific differences in variability should be accounted for when quantifying the deviation from normal EMG patterns. Previously it has been proposed to use the average ensemble of the EMG profile of healthy control subjects as the reference “normal profile” [2]. The rationale for this approach is predicated on the assumption that normal muscle activity patterns across individuals do not differ significantly. However, EMG patterns during walking vary significantly for lower extremity muscles, even within the healthy population [23–25]. Furthermore, previous studies have suggested that some lower extremity muscles have more than one physiologically relevant normal pattern of activity, for example, subjects that have faster walking speeds often have a second period of activity during stance to swing transition for hamstrings; similarly, some subjects have two bursts of gastrocnemius activity during stance which maybe to quickly decelerate tibial rotation at faster walking speeds, while some individuals have single bursts but an earlier activity of gastrocnemius during stance at faster walking speeds. [23, 24]. Healthy individuals tend to have sub-phases of the gait cycle with relatively consistent patterns across subjects, such as consistent “on” or “off” muscle activity. Conversely, certain sub-phases can be extremely variable between subjects. Therefore, rather than pooling the EMG data and identifying an averaged profile composed of significantly different patterns as reference “normal profile”, a tool used for assessment of muscle coordination should account for both the “normal” variability between subjects and between gait sub-phases.

The purpose of the present study was to address the aforementioned limitations of existing quantitative methods by developing a new tool to quantify altered muscle-activation patterns (AMAP). Specifically, our goal is to evaluate deviations between EMG patterns of healthy individuals and those exhibited post-stroke. AMAP accounts for differences in muscle activation patterns associated with altered gait mechanics due to spatiotemporal asymmetries by comparing muscle activity during equivalent gait sub-phases (i.e., first double support, first and second halves of single-leg stance, second double support, and first and second halves of swing). This approach assures that, for example, swing phase activity is not compared to stance phase activity and thus attempts to match the biomechanical demands as closely as possible when comparing muscle activity between healthy and stroke populations. Additionally, we sought to enhance the robustness of detecting “on/off” periods for AMAP by using a *k*-means cluster algorithm. *K*-means can accommodate muscle activity patterns characterized by the lack of a single clear burst of activity and/or increased baseline activity as often occurs post-stroke. Furthermore, since healthy individuals’ muscle patterns tend to be consistent for certain gait sub-phases but extremely variable for some others, AMAP also accounts for normal variability within each sub-phase. Consequently, AMAP is a novel tool that can effectively quantify deficits of motor coordination specifically for hemiparetic gait. The advantages of AMAP would also be relevant to quantifying EMG alterations in other populations.

## Methods

EMG data were collected from eight leg muscles of 34 stroke survivors during treadmill walking at self-selected speeds and 20 healthy individuals at self-selected, 0.3 m/s, 0.6 m/s, and 0.9 m/s walking speeds (see Table 1 for demographic information). All participants provided informed consent approved by the institutional review board. Stroke survivors meeting the following characteristics were included: hemiparesis secondary to a single unilateral stroke; free of significant lower extremity joint pain, range of motion limitations, and profound sensory deficits (including proprioception); ability to ambulate independently –even if requiring an assistive device— over ten meters on a level surface between at least 0.3 m/s but < 1.0 m/s; walk on a daily basis at home; absence of severe perceptual or cognitive deficits; absence of significant lower limb contractures, severe osteoarthritis or prior pathological fracture; and absence of significant cardiovascular impairments contraindicative to walking. Healthy individuals were similarly-aged and were screened for obvious symptoms of neurological disease or lower limb orthopedic impairments.

**Table 1:** Participants’ demographics

		Mean	SD
	Stroke Survivors (*n* = 34)		
Age		61.6	11.8
Time since stroke (mo)		63.3	54.5
Sex (male/female)	25/9		
Side affected (left/right)	18/16		
	Healthy Controls (*n* = 20)		
Age		56.1	9.2
Self-selected walking speed (m/s)		0.95	0.20
Sex (male/female)	11/9		

### Data acquisition

All participants walked on a treadmill for 40 s at their self-selected speeds wearing a safety harness attached to an overhead support to prevent a fall. Healthy controls also walked at 0.3 m/s, 0.6 m/s, and 0.9 m/s, walking speeds. Prior to data collection, subjects walked on the treadmill during an acclimatization session to determine their self-selected speeds. Longer bouts of walking can induce fatigue in stroke survivors, that can influence the gait characteristics [26]. Therefore, to obtain functionally relevant gait data, walking trials of 40 s duration were selected as a trade-off between appropriate number of gait cycles and minimal fatigue. A 16-channel EMG system (MA-416-003 Motion Lab System Baton Rouge, LA) was used to record muscle activity from 8 leg muscles: Tibialis Anterior (TA), Soleus (SO), Medial Gastrocnemius (MG), Vastus Medialis (VM), Rectus Femoris (RF), Lateral Hamstring (LH), Medial Hamstring (MH), Gluteus Medius (GM). Data from both legs of healthy subjects and the paretic leg of stroke subjects are included in the current study. Bilateral ground reaction forces (GRF) were captured via two force plates embedded in the instrumented split-belt treadmill (Bertec Corp., Columbus, OH, USA) and used to identify gait events during treadmill walking. EMG and ground reaction force data were sampled at 2000 Hz except for one subject in whom data were sampled at 1000 Hz. A summary of the stroke survivors’ impairment level and biomechanical characteristics is listed in Table 2.

**Table 2:** Stroke survivors’ characteristics

Subject No.	Self-Selected Walking Speeds (m/s)	Percentage Paretic Propulsion	Lower extremity Fugl-Meyer scores (maximum score = 34)
1	0.30	24.00	11
2	0.50	30.19	16
3	0.50	73.12	17
4	0.65	49.11	21
5	0.40	50.83	18
6	0.60	28.79	19
7	0.35	43.81	18
8	0.40	14.97	8
9	0.35	10.30	8
10	0.75	44.65	15
11	0.30	35.13	15
12	0.45	32.14	14
13	0.50	23.32	16
14	0.40	50.96	15
15	0.50	66.03	20
16	0.40	28.96	15
17	0.60	37.62	22
18	0.45	22.76	12
19	0.45	33.20	11
20	0.35	15.27	12
21	0.45	82.28	20
22	0.50	77.37	20
23	0.35	18.72	13
24	0.30	89.50	22
25	0.55	58.09	21
26	0.40	61.80	21
27	0.50	42.98	17
28	0.90	40.75	21
29	0.30	5.08	12
30	0.40	79.02	21
31	0.35	1.43	17
32	0.40	30.12	15
33	0.25	34.14	12
34	0.30	58.21	13

### Data analysis

EMG data were high pass filtered (20 Hz) with a zero lag fourth-order Butterworth filter, demeaned, rectified, and smoothed with a zero lag fourth-order low-pass (25 Hz) Butterworth filter. The EMG amplitude was normalized to the averaged peak activation across all the steps for each subject to allow comparison between subjects. Since stroke survivors often have difficulty performing isolated voluntary movement, normalizing to maximum voluntary contraction is not a reliable technique for this population. Thus, using peak EMG activation during walking is a widely used normalization technique [27]. Paretic propulsion (Pp) i.e. percentage of the total propulsion generated by the paretic leg was computed by dividing anterior impulse (integral of the positive anterior GRF during walking) of the paretic leg by the sum of the anterior impulse of paretic and non-paretic legs [13].

#### Detecting the “on” and “off” periods of muscle activity

**Fig. 1:**
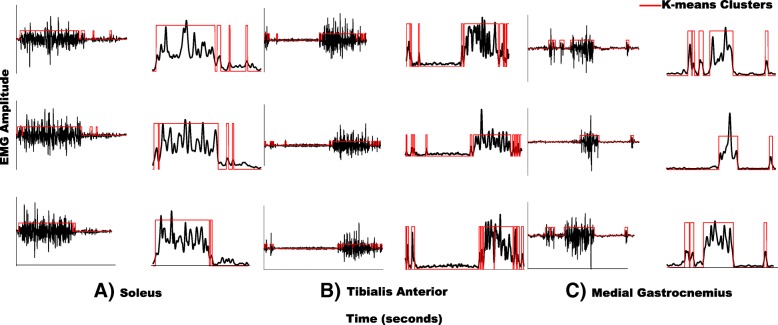
Representative data from stroke survivors’ muscle activity patterns demonstrating the sensitivity of *k*-means cluster analysis to identify “on/off” periods. Each row presents EMG signals for **a**) soleus, **b**) tibialis anterior, and **c**) medial gastrocnemius from stroke survivor’ single gait cycle. Left panels present original EMG data and the right panels present the same representative data after it were high pass filtered (20 Hz) with a zero lag fourth-order Butterworth filter, demeaned, rectified, and smoothed with a zero lag fourth-order low-pass (25 Hz) Butterworth filter. The red solid lines represent *k*-means clusters

To identify muscle activity “on/off” periods during the sub-phases of gait cycle, each point of the linear envelope of each muscle was dichotomized as “on” or “off” using *k*-means cluster analysis with the number of clusters set to five [3]. *K*-means is a data mining method used here to partition the EMG data of each muscle into “*k*” clusters. Data assigned to the cluster with the lowest mean EMG amplitude are assumed to correspond with “off” and data assigned to other clusters classified as muscle activity “on”. The *k*-means cluster analysis has been established for detecting the “on/off” periods within healthy and stroke populations during walking [3, 27, 28]. Furthermore, stroke survivors may have increased baseline muscle activity, and in these cases the *k*-means cluster analysis has shown the ability to differentiate between the increased baseline activity (“off”) and bursts of muscle activity associated with walking (“on”), even when the bursts are short or have a spike-like character. Additionally, in the stroke population, where there is often a lack of single well-defined burst of activity, previous literature investigating the post-stroke population has demonstrated that setting the number of clusters to five is a robust criterion to identify the EMG activity [3, 27]. We also visually inspected the “on/off” timing identified using the *k*-means cluster analysis for the current data set to be confident in the reliability of the method. Figure 1 demonstrates the sensitivity of *k*-means cluster analysis to identify “on/off” periods in EMG data from representative stroke survivor.

#### Characterizing EMG timing and amplitude in the healthy population and identifying the altered patterns of hemiparetic gait

We divided the gait cycle into six regions: first double support (DS1), first (SS1) and second halves of single-leg stance (SS2), second double support (DS2), and first (SW1) and second halves of swing (SW2). In each of the six regions, we calculated timing and amplitude components for each muscle. The timing component was equal to the “on” time within each region expressed as a percentage of the total duration of that region:

1 $$ {Timing\ Component}_{HEALTHY}=\left(\frac{On\  time\ for\ region\kern0.5em }{Total\ time\ for\ region}\right)\ast 100 $$

The amplitude component was equal to the integrated EMG of the “on” time for each region expressed as a percentage of the total integrated EMG of all “on” periods of the gait cycle:

2 $$ {\mathrm{Amplitude}\ \mathrm{Component}}_{HEALTHY}=\left(\frac{\mathrm{Integrated}\ \mathrm{EMG}\ \mathrm{a}\mathrm{mplitude}\ \mathrm{during}\ \mathrm{On}\ \mathrm{time}\ \mathrm{of}\ \mathrm{a}\ \mathrm{region}\kern0.5em }{\mathrm{Total}\ \mathrm{Integrated}\ \mathrm{EMG}\ \mathrm{a}\mathrm{mplitude}\ \mathrm{of}\ \mathrm{the}\ \mathrm{gait}\ \mathrm{cycle}}\right)\ast 100 $$

Equations 1&2 were applied to all healthy subjects and means were computed for the timing and amplitude components in each of the six regions for the eight muscles. As expected, these values have considerable inter-individual variability; therefore, we computed z-scores to address this variability within the healthy population. A z-score is a measure of the number of standard deviations (SD) an element falls above or below the population mean. Defining our sample of healthy individuals as the norm, we used means and SDs of each region/muscle combination for healthy subjects to compute the z-scores within each respective region/muscle.

At their self-selected, as well as 0.3 m/s, 0.6 m/s, and 0.9 m/s, walking speeds healthy individuals exhibited z-scores ranging mostly between ±2.57 for both the amplitude and timing components. This window of ±2.57 corresponds to the 99% confidence interval with less than 9% of healthy controls as outliers (outside of the ±2.57 window) for any muscle/region at all walking speeds. Specifically, at self-selected walking speeds, only 7.6% of healthy controls were outside the ±2.57 window across all regions and muscles. The percentage of the outliers at 0.3 m/s was 6.2%, at 0.6 m/s; 8.2%, and at 0.9 m/s; 7.4%. Additionally, at the self-selected speed, the percent of outliers for each muscle were; SO = 14.1%, MG = 5%, MH = 8.1%, LH = 5.7%, TA = 5.2%, RF = 6.2%, VM = 7.6%, and GM = 8.5% (see Additional file 1 for detailed data and figures). Therefore, we defined the range of ±2.57 as the window for normal patterns, and AMAP scores of stroke survivors falling within the same range indicate agreement with the normative values.

To compare amplitude and timing of each muscle for corresponding regions of stroke survivors’ paretic limb with “normal”, AMAP scores were obtained by comparing the amplitude and timing for each hemiparetic participant with the group mean and SD of healthy individuals (see below). Stroke subjects were stratified into three groups using self- selected walking speeds to classify functional walking ability as described by Perry [29]. Household ambulators (< 0.4 m/s); limited community ambulators (0.4–0.8 m/s); and community ambulators (> 0.8 m/s) were compared to mean and SD for healthy individuals as determined at walking speeds of 0.3 m/s, 0.6 m/s and 0.9 m/s, respectively (see Additional file 1 for details). The AMAP scores of each subject for the timing component of EMG profile were computed for each region as:

3 $$ \mathrm{Timing}\ \mathrm{Component}\ {\mathrm{AMAP}\ \mathrm{Score}}_{SUBJECT}=\frac{\ {\mathrm{Timing}\ \mathrm{Component}}_{SUBJECT}-\mathrm{Timing}\ {\mathrm{Component}\ \mathrm{Mean}}_{HEALTHY}\kern0.5em }{\mathrm{Timing}\ \mathrm{Component}\ {\mathrm{SD}}_{HEALTHY}} $$

We computed the AMAP scores of the amplitude component of muscles within each region as:

4 $$ \mathrm{Amplitue}\ \mathrm{Component}\ {\mathrm{AMAP}\ \mathrm{Score}}_{SUBJECT}=\frac{\ {\mathrm{Amplitude}\ \mathrm{Component}}_{SUBJECT}-\mathrm{Amplitude}\ {\mathrm{Component}\ \mathrm{Mean}}_{HEALTHY}\kern0.5em }{\mathrm{Amplitude}\ \mathrm{Component}\ {\mathrm{SD}}_{HEALTHY}} $$

A negative AMAP score indicates that the muscle has a reduced amplitude or timing component compared to the normal pattern, while a positive value indicates amplitude or timing increases. AMAP can also be used to quantify the overall timing or amplitude patterns of each muscle. We propose to use the absolute values of the AMAP scores for this purpose and then average the scores of all six regions of the gait cycle, thereby obtaining two values for each muscle to assess the deviations in the muscle timing profile or amplitude expressed as:

5 $$ Total\ AMAP\ Score=\frac{1}{n}\sum \limits_{i=1}^n\left( Timing\ or\ {Amplitude\ Component}_i\right) $$

Here “n” represents the number of regions. An AMAP value closer to zero would suggest a stronger agreement with the normal EMG pattern and vice versa. The total AMAP scores for the timing and amplitude components of each muscle for stroke survivors are reported in Additional file 1: Table S3.

#### Statistical analysis

To determine whether AMAP can provide insight into the relationship between altered muscle activity and walking performance in stroke survivors, we tested the correlation of total AMAP scores for the plantarflexors, SO and MG (absolute values of the AMAP scores averaged across six regions), with self-selected walking speed and paretic propulsion (Pp), defined as the percentage of the total propulsion generated by the paretic leg [13]. We performed Pearson’s correlation analyses between Pp and the SO and MG total AMAP scores for the amplitude and timing components. We also performed Pearson’s correlation analyses between self-selected walking speeds and SO and MG total AMAP scores for amplitude and timing components.

## Results

### Altered EMG patterns of stroke survivors identified by AMAP

**Fig. 2:**
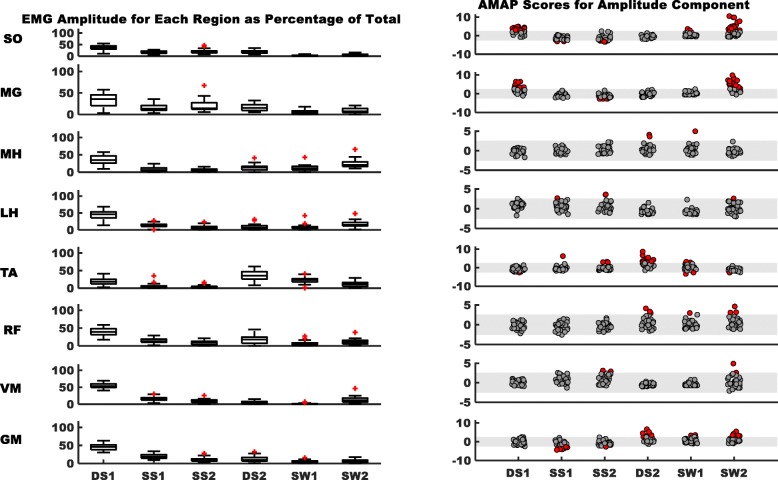
Left panel demonstrates the stroke survivors’ EMG activity amplitude during self-selected walking; the box plots indicate the range in the data, horizontal black line in center is the median, the upper and lower boundaries of the box indicating the upper and lower quartile respectively, and red markers represent extreme values. Right panel demonstrates AMAP scores of stroke survivors for the amplitude component of each muscle. The shaded gray area is the normal range of scores (±2.57). Each dot within a region of gait cycle represents score of a stroke survivor with solid red dots representing the subjects with scores outside the “normal” window of ±2.57. Positive scores, i.e. solid red dots above the “normal” window, represent EMG activation greater than “normal” and vice versa. For the clarity of data, we have adjusted the Y-axes scales on the right panel between ±5 and ± 11. The EMG data from each muscle were normalized to the averaged peak activation across all the steps taken by the subject

**Fig. 3:**
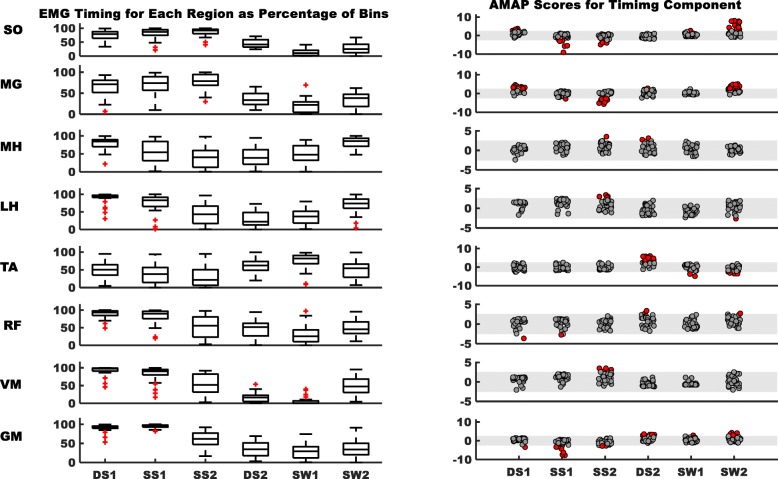
Left panel demonstrates the stroke survivors’ EMG activity timing during self-selected walking; the box plots indicate the range in the data, horizontal black line in center is the median, the upper and lower boundaries of the box indicating the upper and lower quartile respectively, and red marker represent extreme values. Right panel demonstrates AMAP scores of stroke survivors for the timing component of each muscle. The shaded gray area is the normal range of scores (±2.57). Each dot within a region of gait cycle represents score of a stroke survivor with solid red dots representing the subjects with scores outside the “normal” window of ±2.57. Positive scores represent duration of EMG activation longer than “normal” and vice versa. For the clarity of data, we have adjusted the Y-axes scales on the right panel between ±5 and ± 11. The EMG data from each muscle were normalized to the averaged peak activation across all the steps taken by the subject

**Fig. 4:**
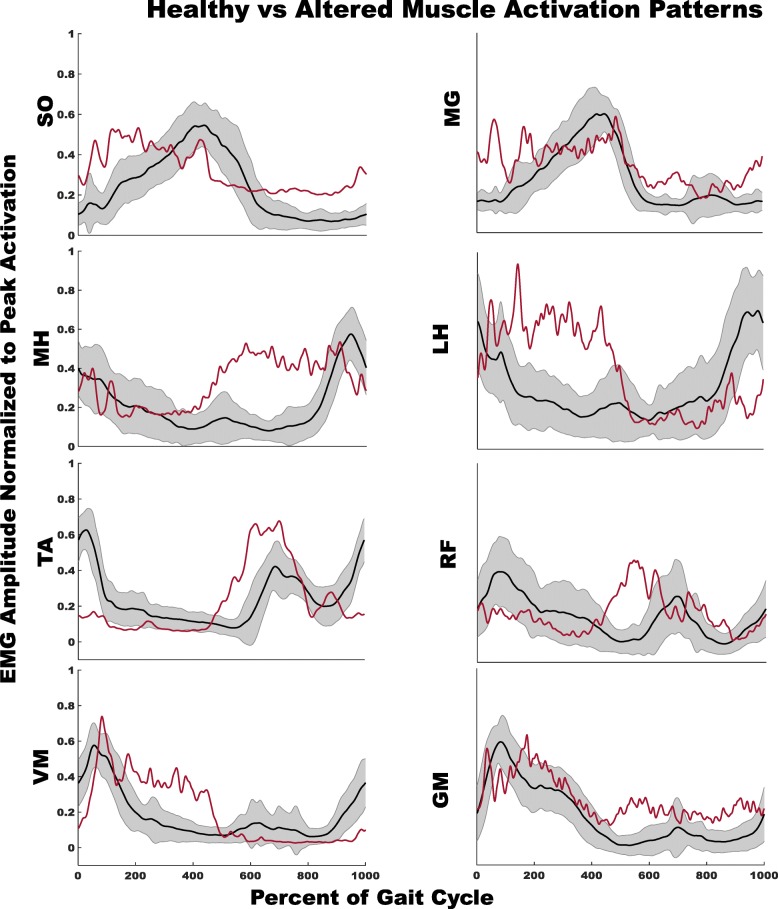
Examples of altered EMG patterns in comparison to normal pattern are illustrated for EMG activity at self-selected walking speeds for all muscles. Black solid lines are the mean EMG activity of healthy controls and shaded grey area is the SD. Red solid lines are EMG activity of a representative stroke survivor with Table 3 representing the corresponding AMAP scores for the amplitude and timing components (values highlighted in red in the table represent scores outside the “normal” window of ±2.57). To clearly present the EMG activity, time is presented as percent of gait cycle

The current method identified altered EMG patterns during walking in stroke survivors, where post-stroke subjects tend to have positive AMAP scores for timing and amplitude during sub-phases in which the muscle is typically “off”, and negative scores during the gait cycle sub-phases that normally demonstrate consistent “on” activity (Figs. 2 and 3 right panels, red dots for each bar represent the stroke survivors outside the “normal” widow within each region of every muscle). Figure 4 demonstrates deviations in the activation patterns of all muscles in representative stroke survivor and Table 3 presents the corresponding AMAP scores of each muscle for the representative stroke survivor. Specifically, 79% of subjects for the amplitude component and 73% for the timing for SO had altered patterns (i.e., total number of subjects outside the “typical” range for healthy controls). MG patterns showed 62 and 79% of subjects with altered patterns within the amplitude and timing components, respectively. Most of these alterations for SO and MG demonstrated increased “on” time and amplitude during DS1 and SW2 and decreased “on” time and amplitude during SS2. TA demonstrated 62 and 59% subjects with altered amplitude and timing components, respectively, with most of the alterations presented as increased amplitude and “on” time during DS2. 48% subjects for amplitude and 51% for the timing component for GM had altered patterns with most of the alterations having an increased amplitude and “on” time during DS2 and SW2, and a decreased amplitude and “on” time during SS1.

**Table 3:** Total amap scores for representative stroke survivor in Fig. 4 (values highlighted in red represent scores outside the “normal” window of ±2.57)

	Region1	Region2	Region3	Region4	Region5	Region6
Average and SD Total AMAP Scores for Amplitude Component						
SO	2.89	0.88	1.24	1.68	1.00	4.07
MG	2.80	0.58	1.97	0.34	0.40	3.38
MH	1.47	0.78	0.24	4.12	1.82	0.88
LH	0.17	2.29	3.55	0.81	1.27	1.15
TA	2.04	1.66	1.41	4.63	0.28	0.89
RF	1.59	2.17	1.16	4.14	1.08	0.26
VM	0.83	2.57	3.14	0.70	0.69	2.10
GM	1.11	2.49	1.71	2.91	1.62	3.07
Average and SD Total AMAP Scores for Timing Component						
SO	2.06	0.35	0.22	1.48	0.95	3.51
MG	3.62	0.39	0.66	2.06	0.53	3.57
MH	1.11	0.31	0.46	3.13	2.29	0.14
LH	1.49	2.41	3.39	0.60	1.66	1.72
TA	1.90	1.73	1.46	4.71	0.09	0.69
RF	0.48	1.28	0.96	2.31	1.12	0.05
VM	0.07	1.97	3.50	0.80	0.72	2.02
GM	0.46	2.74	1.64	2.31	0.95	2.08

The percentage of subjects with altered patterns within the amplitude and timing components was relatively smaller for each of the remaining four muscles. Specifically, the total number of subjects outside the “typical” range in healthy controls for the following muscles: MH 11%; 11%, LH 13%; 16%, RF 24%; 14%, and VM 12%; 15% had altered patterns for the amplitude and timing component respectively (although note that these occurrences greatly exceed those seen in the control subjects). Therefore, to determine whether there might be substantial deviations not captured by AMAP for MH, LH, RF, and VM, we performed a post-hoc analysis for the aforementioned muscle patterns’ AMAP scores. We narrowed the window from ±2.57 to ±2.05 (corresponding to changing from 99 to 96% confidence interval). The narrow window resulted in the number of subjects outside the “normal” window for MH 21%; 28%, LH 20%; 36%, RF 31%; 14%, and VM 32%; 44% for the amplitude and timing components, respectively. Most of the additional subjects with the narrower window for quadriceps were seen during SS2 and for hamstrings during SS1 and SS2 with increased activity. For comparisons with normal variability at a narrower window, we performed a similar post-hoc analysis (narrower window from ±2.57 to ±2.05) for MH, LH, RF, and VM in healthy individuals at their self-selected walking speed. The percentage of the outliers for healthy individuals with a window of ±2.57 was MH 8%; 8%, LH 11%; 6%, RF 12%; 0%, and VM 9%; 6% for amplitude and timing components respectively. This changed to MH 16%; 16%, LH 29%; 17%, RF 19%; 13%, and VM 21%; 27% with a window of ±2.05. By using a less conservative window for stroke survivors and healthy controls we can compare the additional outliers between the two populations and evaluate whether the additional outliers in stroke population are a result of altered patterns or normal variability.

### Altered muscle patterns and their relationship with gait biomechanics and speed

**Fig. 5:**
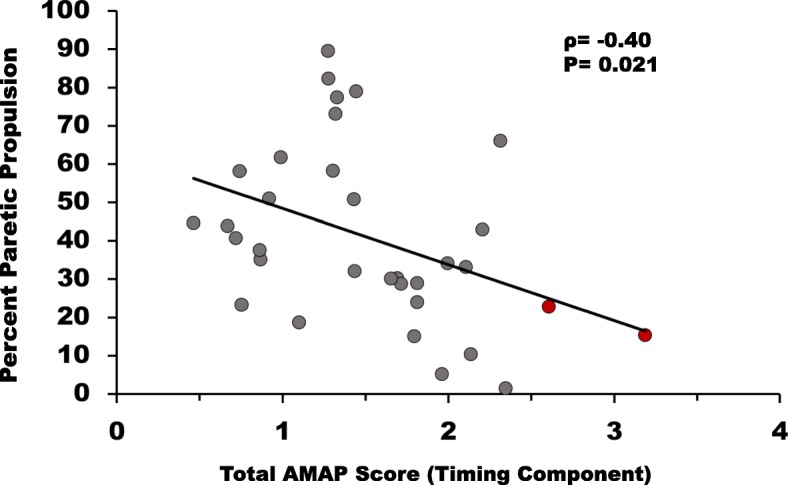
Correlations between Pp and the total AMAP scores for SO averaged across all regions of the timing component. The total AMAP scores were negatively associated (*p* < 0.05) with Pp. The red dots represent stroke subjects that had AMAP scores outside of the “normal” window of ±2.57

**Fig. 6:**
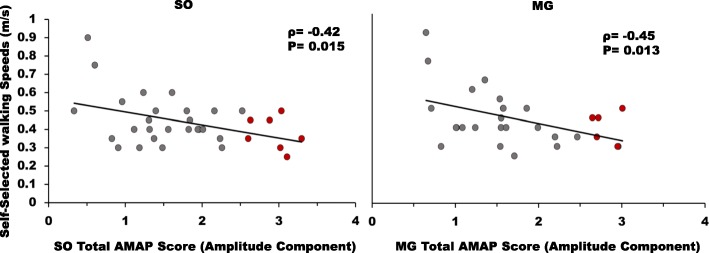
Correlations between self-selected walking speeds and total AMAP scores for the amplitude components (scores averaged across all regions of the amplitude component) of SO (left panel) and MG (right panel). The red dots represent stroke subjects that had AMAP scores outside of the “normal” window of ±2.57. The total AMAP scores of SO and MG for amplitude were negatively associated with the walking ability of stroke survivors (p < 0.05)

The total AMAP score for the timing component for stroke survivors’ SO muscle was negatively correlated (*p* = 0.02) with Pp (Fig. 5), and a tendency towards a negative relationship was observed between SO amplitude component and Pp (*p* = 0.05). We did not find a significant correlation between Pp and MG timing (*p* = 0.72) or amplitude (*p* = 0.22) components. The total AMAP scores for the amplitude component of SO (*p* = 0.01) and MG (*p* = 0.01) were negatively correlated with their self-selected walking speeds (Fig. 6). A significant negative correlation was also observed between the total AMAP score for the SO timing component (*p* = 0.03), however there was no correlation between the timing component of the total AMAP for MG (*p* = 0.13) with walking speeds.

## Discussion

The current study developed a new tool, the AMAP, to quantify altered muscle activation patterns during walking thus enabling evaluation of deviations between EMG patterns of healthy individuals and stroke survivors. AMAP demonstrates the ability to quantify alterations in timing and amplitude of EMG patterns during walking while accounting for the normal variability among healthy individuals and adjusting for gait asymmetries thus attempting to compare biomechanically similar events. In the current study, we defined the normal range of AMAP for healthy subjects as z-scores within ±2.57 (i.e., the 99% confidence interval). For the stroke population AMAP scores within this window were considered normal while scores outside the window were identified as altered. The alterations in the stroke population quantified using AMAP, agree with previously reported, mostly qualitatively identified, EMG alterations observed in gait patterns following stroke [9, 11, 30, 31]. Thus, our results suggest, AMAP can be successfully used as a tool to compare muscle activity for similar regions of the gait cycle and determine the relationship between altered muscle activity and the corresponding biomechanical deviations within the stroke population.

### AMAP and existing tools for quantitative assessment of muscle activity

While some previous studies have proposed methods for quantifying EMG patterns during walking, the AMAP addresses their limitations for quantifying altered EMG patterns in hemiparetic gait. Previously, Ricamato et al. [1] proposed a method to quantify deviations of EMG patterns from normal, using a single value between zero and one, such that the values closer to one are interpreted as ‘better’ in comparison to smaller values. Erni and Colombo [3], proposed to identify the similarities or differences between normal and pathological gait by examining correlation and relative variability of EMG patterns. However, these methods do not account for temporal asymmetries in walking, typically lack a robust method of identifying EMG “on/off” activity, and do not account for inter-individual variability within the healthy population. Although, Hof et al. [2] have acknowledged inter-individual variability by identifying high and low limits based on healthy data as the range of normal profile, but their approach does not account for potential spatiotemporal asymmetries in pathological gait. Additionally, Den Otter et al. 2007 [3] identified EMG “on/off” activity using *k*-means cluster analysis within each sub-phase of gait cycle; however, they did not account for “normal” variability within the healthy population. Therefore, each of these methods had at least one of the aforementioned limitations, which would limit their accuracy to identify EMG alterations in the stroke population. We believe that our approach, to collectively use a more robust method for identifying the “on/off” muscle activity periods, along with accounting for asymmetric gait post-stroke and inter-individual variability within the reference healthy population to compute AMAP scores, offers improvement over previously proposed methods for identifying deviations of the EMG pattern during walking. However, future studies are needed to make statistical comparisons of performance between previously proposed methods and AMAP for identifying deviations of the EMG patterns during walking.

### AMAP as a tool to identify the alterations of muscle activity following stroke

The alterations in the EMG patterns within the stroke population identified by AMAP are consistent with the exiting literature reporting the typical qualitatively identified altered EMG patterns. In the current study, stroke survivors demonstrated the largest alterations in SO, MG, TA, and GM muscles with these altered patterns consistent with previous literature on stroke gait [9, 11, 30, 31]. The plantarflexors demonstrated a tendency for increased timing and amplitude AMAP scores during DS1 and SW2. The increased activity of plantarflexors during early stance is often seen in the stroke population [9, 11]. Furthermore, it has been suggested that due to co-activation some stroke survivors may also have increased plantarflexor activity during the end of swing phase [9]. Subjects also demonstrated decreased timing and amplitude AMAP scores during SS2, which may suggest weak plantarflexors post-stroke [9]. Some of the stroke survivors showed increased TA AMAP scores for timing and amplitude during DS2, which may be due to increased ankle muscle co-activation observed in hemiparetic gait [30]. Increased GM timing and amplitude AMAP scores during DS2 and SW2 may be present to maintain gait stability because stroke survivors with poor gait stability often demonstrate an increase in paretic GM activity during swing associated with increased mediolateral center of mass velocity towards the non-paretic limb [31]. Furthermore, decreased GM timing and amplitude AMAP scores during SS1 could be due to insufficient muscle activity and weakness [9]. However, one limitation of the current tool is that since the amplitude scores were defined as a percentage of cycle, the scores are influenced by the total “on” time of EMG activity. Therefore, we urge caution when interpreting the amplitude scores.

Altered patterns for other muscles were relatively subtle. One reason for fewer alterations is larger inter-individual variability for MH, LH, RF, and VM within the healthy population, which may be more pronounced when walking at slower speeds (e.g., at 0.3 or 0.6 m/sec). However, in healthy individuals, biomechanical demands vary with change in walking speeds, i.e. changes in joint angles, joint moments and spatiotemporal parameters such as stance duration and stride length are associated with change is walking speeds [32]. Therefore, we expect that we have improved the results of our analysis by matching the speed of the control profile to that of the hemiparetic profile. We believe speed dependent changes should be controlled for, as they do not represent an effect of the hemiparesis. However, post-hoc analysis with a narrower “normal” window increased the number of stroke survivors outside the “normal” window for MH, LH, RF, and VM patterns. The additional stroke survivors outside the “normal” window for quadriceps were seen with increased activity during SS2, also observed sometimes in “normal” patterns [9]. The additional stroke survivors for hamstrings had increased activity during SS1 and SS2, seen sometimes in healthy individuals [24]. Furthermore, the post-hoc analysis in healthy data revealed that the number of healthy individuals outside the narrower window in comparison to a window of ±2.57, showed similar trends of change as stroke data (for example, for MH both the healthy and stroke populations demonstrated that the subjects outside the narrower window were twice the number of their subjects at wider window). Therefore, we believe that a window of ±2.57 is a conservative estimate of an altered pattern as narrowing the window does not seem to preferentially result in the identification of more altered patterns in the post-stroke population. This suggests that the activation patterns for MH, LH, RF, and VM in the current cohort of stroke survivors are more similar to healthy patterns compared to other muscles. Furthermore, Knutsson et al. demonstrated that some of the stroke survivors had hamstrings and quadriceps patterns similar to normal [9], and, contributions primarily from the SO, MG, and GM of the paretic leg have been associated with the functional walking status of stroke survivors [14]. Therefore, we believe that the AMAP was successful in quantitatively identifying deviations of the muscle patterns during walking within the stroke population. Although, we did not include the EMG data of the non-paretic leg in the current study, given the temporal asymmetries of both paretic and non-paretic leg during walking, AMAP would be an ideal tool to identify altered muscle activation patterns for the non-paretic leg in stroke population.

### Can AMAP be used to understand the relationship between altered EMG and biomechanical patterns following stroke?

We propose that AMAP is useful for identifying and interpreting relationships between altered muscle activity and biomechanical measures, and may aid clinicians in designing a protocol that may improve biomechanical characteristics within the stroke population. AMAP can be used in multiple ways to evaluate alterations of EMG patterns. For example, we envision the primary use to be independently examining AMAP scores of each muscle for each sub-phase of the gait cycle. However, one could also compute total AMAP scores (i.e. the absolute scores averaged across all the sub-phases for an individual muscle or even of all of the muscles). Phase-specific evaluation may provide detailed understanding of muscle activities and the corresponding biomechanical responses within every sub-phase. Total AMAP scores represent the average of each sub-phase score that accounts for spatiotemporal asymmetries, therefore total AMAP scores represent EMG alterations during the gait cycle. A phase-by-phase comparison of EMG alterations with the analogous alterations of biomechanical responses for each muscle is beyond the scope of this study (however see Table 2 and Additional file 1 for details on AMAP scores, Pp, and self-selected walking speeds of stroke survivors). Nevertheless, as an example of the utility of the method, we used it to identify the relationship between Pp and self-selected walking speeds of stroke survivors and their total AMAP scores for plantarflexor muscles. Current results are a proof of concept for future experiments to use AMAP as a tool to identify specific muscle activation patterns that should be targeted by clinicians to improve specific biomechanical responses during gait rehabilitation. In the current study, Pp was negatively correlated with the SO total AMAP scores for the timing component and a trend towards a negative correlation was also observed between Pp and the amplitude component. This suggests that an activation pattern of SO closer to normal, is associated with greater Pp; however, timing of SO activation maybe more important than the amplitude. Total AMAP scores of the amplitude component for SO and MG were negatively correlated with gait speed. This observation suggests that improving the amplitude pattern of plantarflexors towards normal may be important for improvement in gait speed, however future studies are needed to further understand the relationship between altered plantarflexor coordination and Pp or walking speed in stroke population.

AMAP can also be applied to assess the effects of gait rehabilitation targeted to improve altered patterns of the desired muscles (e.g., interventions attempting to restore more normal timing and amplitude of activity). As discussed earlier, the primary impairment of locomotion post-stroke is reduced ability to sufficiently recruit and appropriately time muscle activity. AMAP will allow comparison of different conditions of gait training to determine which condition results in the “best” achievable activation pattern. In future studies AMAP can be applied to understand the effects of gait training parameters in the stroke population. For example, the AMAP can be used to identify an efficient and effective combination of speed, weight support and therapists’ assistance to improve muscle amplitude and timing patterns during walking following body weight supported treadmill training.

### Limitations of the proposed tool

Although AMAP appears to be a useful tool for evaluating deviations of EMG patterns in gait following stroke, there are certain considerations when applying this method. First, the window defined as “normal” is based on the inter-individual variability within a cohort of healthy population for a specific walking activity. However, when walking tasks change too much, these values are not likely appropriate to represent the normal range of EMG patterns. Therefore, to identify the reference “normal” pattern for different walking conditions, data from healthy individuals during those walking conditions should be acquired for comparison (e.g., when walking at significantly different speeds or levels of body weight support). Note that we compared data from post-stroke subjects to control data collected at a similar walking speed (e.g., at 0.3, 0.6, or 0.9 m/sec), rather than at self-selected speed, which differs greatly between post-stroke and healthy populations. Second, due to the difficulty of obtaining meaningful measures of maximal voluntary contraction or comparing absolute magnitude of EMG activation in millivolts across subjects, the magnitude measure is scaled relative to observations made during walking and not related to an absolute known quantity. Thus, we cannot differentiate between muscles activated to an “appropriate” level and those deemed under- or over-active during hemiparetic gait.

## Conclusion

We propose a method to effectively quantify the deviations of muscle activity during walking specifically within the stroke population. This quantitative analysis can be used to identify the gait phases that may require more attention than others when developing an optimal training paradigm. It is by no means intended to replace existing methods for evaluating coordination patterns during walking, but to provide a quantitative measure that can be used in conjunction with other kinetic and kinematic measures to enhance the current understanding of muscle coordination during walking. Normal muscle activation quality measures were established in similarly aged subjects walking at speeds matched to the stroke survivors’ self-selected walking speeds. Future studies can extend the AMAP technique to identify the alterations of pathological muscle patterns under conditions other than stroke gait at self-selected speeds if the appropriate normal population is sampled performing the desired walking task (e.g., walking at different speeds, different body weight support conditions, etc.).

## Additional file

Additional file 1: **Figure S1.** AMAP scores for all healthy individuals at all four walking speeds are provided. **Figure S2.** Average and SD of healthy individuals’ EMG patterns at all walking speeds. **Table S1.** EMG patterns for healthy individuals at all four walking speeds. **Table S2.** EMG patterns for stroke survivors at self-selected walking speeds. **Table S3.** Total AMAP scores for stroke survivors at their self-selected walking speeds (ZIP 1538 kb)

## Abbreviations

AMAP: Altered muscle activation patterns
DS1: First double support
DS2: Second double support
EMG: Electromyography
GM: Gluteus Medius
GRF: Ground reaction force
LH: Lateral Hamstring
MG: Medial Gastrocnemius
MH: Medial Hamstring
Pp: Paretic propulsion
RF: Rectus Femoris
SD: Standard deviations
SO: Soleus
SS1: First halve of single-leg stance
SS2: Second halve of single-leg stance
SW1: First halves of swing
SW2: Second halves of swing
TA: Tibialis Anterior
VM: Vastus Medialis
