# Supplementary material for: Altered muscle activation patterns (AMAP): an analytical tool to compare muscle activity patterns of hemiparetic gait with a normative profile
Source: J Neuroeng Rehabil. 2019 Jan 31;16:21. doi: 10.1186/s12984-019-0487-y (PMC6357420; doi:10.1186/s12984-019-0487-y)
Supplement: Supplementary file 1 — Figure S1. AMAP scores for all healthy individuals at all four walking speeds are provided. Figure S2. Average and SD of healthy individuals’ EMG patterns at all walking speeds. Table S1. EMG patterns for healthy individuals at all four walking speeds. Table S2. EMG patterns for stroke survivors at self-selected walking speeds. Table S3. Total AMAP scores for stroke survivors at their self-selected walking speeds (ZIP 1538 kb) [file 12984_2019_487_MOESM1_ESM.zip › Supplimentry Data_Tables_Final.pdf]

Table S1. EMG PATTERNS FOR HEALTHY INDIVIDUALS AT FOUR DIFFERENT WALKING SPEEDS

Gait Speed 0.3 m/s						
	**Region1**	**Region2**	**Region3**	**Region4**	**Region5**	**Region6**
**EMG Amplitude as Percent of Gait Cycle**						
**%SO Mean±SD**	24.63±6.71	23.58±5.96	22.94±4.56	26.97±9.98	0.81±2.22	0.91±1.45
**%MG Mean±SD**	18.97±8.64	19.89±7.12	30.43±12.69	26.53±10.60	2.96±5.29	1.04±2.01
**%MH Mean±SD**	44.02±18.98	7.69±7.17	3.77±4.94	12.78±10.78	10.68±8.90	20.96±11.72
**%LH Mean±SD**	36.04±12.79	7.99±5.57	4.62±4.15	22.38±15.02	16.36±11.22	12.50±5.54
**%TA Mean±SD**	27.94±11.16	7.71±4.54	5.42±3.84	20.93±8.81	19.80±6.69	18.07±7.93
**%RF Mean±SD**	43.89±13.09	18.41±5.21	12.11±5.84	14.19±8.32	5.55±5.32	5.71±3.87
**%VM Mean±SD**	55.30±13.38	11.37±5.83	5.27±3.76	14.70±13.92	3.30±4.70	9.98±5.83
**%GM Mean±SD**	46.82±6.43	24.93±3.58	16.56±4.88	6.29±4.12	2.86±2.64	2.40±2.67
**EMG Activity Timing as Percent of Activity Timing Within Each Region**						
**%SO Mean±SD**	53.44±15.94	91.82±7.73	90.83±10.34	54.50±16.42	5.81±13.21	6.13±7.77
**%MG Mean±SD**	33.44±15.03	68.82±21.96	79.45±14.99	35.84±11.27	15.77±26.38	6.64±11.11
**%MH Mean±SD**	70.07±23.31	36.07±29.61	20.42±23.86	28.79±21.20	47.36±28.03	70.44±20.07
**%LH Mean±SD**	65.94±20.86	41.21±26.78	26.27±23.67	39.26±20.45	60.70±25.67	54.66±19.12
**%TA Mean±SD**	49.89±17.35	50.19±27.93	39.27±26.74	32.03±11.67	73.24±16.65	67.69±19.09
**%RF Mean±SD**	81.76±13.37	81.16±13.44	60.18±24.94	32.46±18.26	30.18±27.29	31.89±19.57
**%VM Mean±SD**	75.99±19.23	52.32±25.26	28.93±22.64	25.85±19.94	15.71±21.23	40.28±21.68
**%GM Mean±SD**	84.31±9.06	99.05±2.19	86.37±16.43	19.87±13.78	22.44±19.77	17.38±15.72

Gait Speed 0.6 m/s						
	**Region1**	**Region2**	**Region3**	**Region4**	**Region5**	**Region6**
**EMG Amplitude as Percent of Gait Cycle**						
**%SO Mean±SD**	14.97±5.69	25.28±5.59	34.10±5.88	23.27±8.65	1.23±2.57	0.94±1.69
**%MG Mean±SD**	9.68±5.88	26.37±10.73	46.67±12.07	12.30±8.85	3.35±6.04	1.40±2.40
**%MH Mean±SD**	37.88±16.45	10.46±9.96	4.14±5.26	7.95±8.02	7.69±7.04	31.76±14.43
**%LH Mean±SD**	31.16±12.89	9.49±7.91	5.61±5.08	12.54±10.13	17.74±11.77	23.30±13.19
**%TA Mean±SD**	28.88±9.10	6.61±4.60	3.99±3.44	15.61±5.21	23.03±6.59	21.75±7.75
**%RF Mean±SD**	41.04±10.78	19.34±6.78	9.81±7.09	12.85±8.09	8.08±7.70	8.74±6.36
**%VM Mean±SD**	52.20±14.02	12.57±6.93	5.31±6.43	11.18±12.78	3.83±5.52	14.81±6.47
**%GM Mean±SD**	46.87±6.92	29.20±4.59	13.10±5.74	4.01±4.31	3.53±3.45	3.12±3.39
**EMG Activity Timing as Percent of Activity Timing Within Each Region**						
**%SO Mean±SD**	43.69±14.79	87.32±10.20	95.41±7.32	52.99±16.33	7.95±15.32	5.52±7.21
**%MG Mean±SD**	24.52±14.95	72.87±22.07	92.39±7.24	25.82±14.28	15.20±23.18	6.85±10.16
**%MH Mean±SD**	70.62±20.25	38.10±32.01	18.48±22.38	24.50±22.33	31.23±25.15	78.40±19.53
**%LH Mean±SD**	68.94±17.79	39.84±23.64	24.44±19.76	32.25±20.58	57.72±26.28	69.23±19.84
**%TA Mean±SD**	57.32±17.24	34.64±22.23	26.14±23.03	31.81±10.22	76.82±13.75	69.46±16.87
**%RF Mean±SD**	88.47±10.84	74.58±19.93	43.54±26.33	33.62±18.72	33.75±26.96	37.55±23.56
**%VM Mean±SD**	85.44±14.43	48.70±24.90	22.50±19.73	26.51±23.17	17.05±23.56	50.29±21.64
**%GM Mean±SD**	89.76±6.59	97.44±3.42	61.97±21.94	15.14±15.93	20.70±18.52	17.51±17.29

Gait Speed 0.9 m/s						
	**Region1**	**Region2**	**Region3**	**Region4**	**Region5**	**Region6**
**EMG Amplitude as Percent of Gait Cycle**						
**%SO Mean±SD**	11.66±5.13	24.89±6.46	39.62±6.94	21.26±7.70	1.49±2.94	0.83±0.98
**%MG Mean±SD**	6.09±4.90	28.96±10.87	47.99±8.02	10.80±8.15	3.88±6.26	2.01±3.24
**%MH Mean±SD**	31.71±13.02	9.31±9.64	4.30±4.76	5.28±4.71	5.92±6.55	43.37±17.19
**%LH Mean±SD**	30.87±12.14	7.92±6.87	6.13±7.08	6.46±5.41	14.85±13.05	33.63±12.77
**%TA Mean±SD**	31.79±8.12	6.68±4.61	3.00±3.00	12.14±4.26	23.52±5.94	22.72±7.73
**%RF Mean±SD**	42.37±13.03	16.29±7.87	7.02±6.53	13.45±8.43	11.78±8.67	8.92±6.69
**%VM Mean±SD**	57.42±13.60	8.55±6.34	2.88±3.44	9.86±12.68	4.17±6.30	17.04±8.23
**%GM Mean±SD**	46.90±8.47	29.48±5.82	9.66±6.24	3.55±4.41	5.65±6.13	4.60±3.93
**EMG Activity Timing as Percent of Activity Timing Within Each Region**						
**%SO Mean±SD**	41.70±15.56	85.12±13.06	97.76±3.47	53.50±16.60	8.59±15.06	5.27±6.23
**%MG Mean±SD**	20.24±14.90	76.49±21.09	95.24±5.97	27.35±14.60	16.08±21.19	9.86±13.99
**%MH Mean±SD**	66.73±20.92	32.81±29.21	19.00±21.15	19.89±16.49	23.33±23.42	85.63±12.95
**%LH Mean±SD**	71.88±14.81	30.64±21.43	22.64±18.35	20.51±15.65	45.86±30.60	80.16±17.52
**%TA Mean±SD**	67.08±13.19	30.41±20.55	17.46±17.12	29.99±9.01	80.02±10.12	71.97±15.76
**%RF Mean±SD**	91.32±5.44	60.17±26.22	30.02±25.57	36.21±21.55	38.56±24.46	33.80±20.65
**%VM Mean±SD**	89.10±8.70	32.72±23.14	13.48±16.40	25.87±27.13	14.77±19.29	49.54±20.34
**%GM Mean±SD**	92.88±5.55	94.26±6.42	42.02±22.73	15.63±18.59	25.31±21.97	22.81±18.93

Self-Selected Speed						
	**Region1**	**Region2**	**Region3**	**Region4**	**Region5**	**Region6**
**EMG Amplitude as Percent of Gait Cycle**						
**%SO Mean±SD**	11.81±7.88	24.85±6.33	40.89±10.74	19.54±9.03	1.37±2.46	1.29±1.83
**%MG Mean±SD**	6.32±6.08	27.15±10.67	49.47±10.03	10.54±9.36	3.83±7.01	2.41±3.62
**%MH Mean±SD**	28.40±12.91	9.46±8.76	4.44±5.02	5.85±6.55	6.61±6.16	45.11±16.07
**%LH Mean±SD**	28.75±11.67	8.51±7.23	6.10±6.52	6.90±7.67	10.60±9.89	39.00±13.89
**%TA Mean±SD**	30.56±8.05	6.06±3.93	2.56±2.65	13.69±5.44	24.08±5.67	22.87±6.64
**%RF Mean±SD**	39.47±10.77	17.42±10.21	7.56±6.66	14.98±12.06	11.99±11.10	8.40±7.28
**%VM Mean±SD**	57.67±13.67	11.28±7.80	2.71±3.40	7.35±8.78	3.63±6.97	17.25±7.98
**%GM Mean±SD**	47.86±8.92	28.96±5.72	8.48±5.72	3.52±4.38	5.49±6.58	5.52±4.61
**EMG Activity Timing as Percent of Activity Timing Within Each Region**						
**%SO Mean±SD**	41.45±19.64	84.27±16.59	96.40±5.80	49.91±20.74	8.64±14.44	7.07±8.31
**%MG Mean±SD**	19.67±16.99	72.95±22.61	93.93±7.27	25.72±15.30	14.39±21.28	11.10±14.46
**%MH Mean±SD**	68.13±22.79	34.72±30.11	20.09±22.49	21.79±19.53	26.27±20.60	90.28±12.10
**%LH Mean±SD**	73.26±17.75	33.07±23.08	24.41±20.97	22.82±17.64	38.61±30.60	85.13±11.29
**%TA Mean±SD**	66.97±14.07	28.22±18.83	15.22±15.89	33.99±10.75	82.22±12.07	73.81±16.21
**%RF Mean±SD**	89.77±9.23	60.37±31.47	30.95±25.97	36.42±20.32	34.62±22.07	31.06±17.73
**%VM Mean±SD**	92.04±6.49	39.83±25.42	13.24±17.05	22.35±24.71	12.89±17.62	51.49±19.09
**%GM Mean±SD**	93.57±6.21	91.85±10.60	37.03±22.16	15.38±19.60	24.65±21.84	25.14±20.23

Table S2. EMG PATTERNS FOR STROKE SURVIVORS AT SELF-SELECTED WALKING SPEEDS

	Stroke Survivors					
	**Region1**	**Region2**	**Region3**	**Region4**	**Region5**	**Region6**
**EMG Amplitude as Percent of Gait Cycle**						
**%SO Mean±SD**	35.57±10.95	16.64±5.95	19.72±9.21	19.42±7.04	2.52±2.78	5.94±4.99
**%MG Mean±SD**	33.06±14.17	15.62±8.37	20.75±13.62	16.98±8.90	4.81±4.53	8.59±5.52
**%MH Mean±SD**	36.44±13.06	8.47±6.08	5.47±4.44	13.08±8.96	11.78±8.64	24.62±12.20
**%LH Mean±SD**	44.71±11.02	12.96±6.62	6.99±5.70	8.98±8.32	8.18±7.78	18.03±10.79
**%TA Mean±SD**	19.50±10.29	5.63±6.54	3.93±4.29	36.23±13.92	23.19±9.45	11.34±6.62
**%RF Mean±SD**	39.79±10.40	15.24±7.22	8.37±5.73	18.01±12.52	7.26±7.65	11.16±7.60
**%VM Mean±SD**	54.72±7.83	16.72±6.35	8.68±5.23	5.55±4.59	1.06±1.80	13.14±9.16
**%GM Mean±SD**	45.99±8.73	20.00±6.84	10.05±5.68	11.78±7.34	5.13±3.69	6.86±4.74
**EMG Activity Timing as Percent of Activity Timing Within Each Region**						
**%SO Mean±SD**	74.59±16.93	80.64±19.66	84.81±18.12	45.30±14.71	14.01±13.28	29.22±21.42
**%MG Mean±SD**	63.43±21.58	70.26±24.65	77.86±19.82	36.06±15.54	19.92±17.21	35.01±17.61
**%MH Mean±SD**	79.09±17.41	53.94±30.48	38.28±27.82	41.20±25.22	50.17±25.84	81.59±15.46
**%LH Mean±SD**	88.90±16.64	75.14±25.21	45.34±28.95	28.11±20.80	37.76±20.03	70.10±23.46
**%TA Mean±SD**	50.99±22.96	37.82±26.75	30.71±28.49	63.84±20.16	74.14±23.27	49.61±24.21
**%RF Mean±SD**	89.26±12.13	81.69±21.45	52.20±30.27	46.68±24.08	31.59±27.14	50.60±22.76
**%VM Mean±SD**	92.43±12.12	82.82±20.87	54.02±27.71	16.15±13.16	6.30±10.53	50.65±24.74
**%GM Mean±SD**	90.55±9.24	94.19±5.26	60.21±19.67	36.62±18.96	29.29±19.55	36.13±22.61

Table S3. TOTAL AMAP SCORES FOR STROKE SURVIVORS AT THEIR SELF-SELECTED WALKING SPEEDS

	**Region1**	**Region2**	**Region3**	**Region4**	**Region5**	**Region6**
**Average and SD Total AMAP Scores for Amplitude Component**						
**SO Mean±SD**	2.60±1.31	1.37±0.94	1.98±1.05	0.86±0.51	0.89±0.98	3.40±3.05
**MG Mean±SD**	2.60±1.67	1.11±0.62	1.52±0.69	0.99±0.59	0.69±0.52	3.46±2.64
**MH Mean±SD**	0.58±0.42	0.58±0.35	0.73±0.53	0.87±0.96	0.85±0.94	0.63±0.49
**LH Mean±SD**	1.00±0.62	0.96±0.68	0.96±0.91	0.86±0.39	0.93±0.43	0.86±0.76
**TA Mean±SD**	1.16±0.68	1.08±1.03	0.91±0.80	2.73±1.83	1.11±0.97	1.15±0.68
**RF Mean±SD**	0.77±0.47	1.07±0.73	0.75±0.54	1.27±0.95	0.84±0.65	1.16±1.05
**VM Mean±SD**	0.47±0.29	0.94±0.71	0.99±0.89	0.58±0.29	0.58±0.19	1.08±0.95
**GM Mean±SD**	1.05±0.78	2.00±1.26	1.36±0.64	1.70±1.56	1.05±0.89	1.64±1.42
**Average and SD Total AMAP Scores for Timing Component**						
**SO Mean±SD**	1.81±0.91	1.58±1.95	1.31±1.97	0.88±0.56	0.79±0.71	3.32±2.61
**MG Mean±SD**	2.36±1.24	0.90±0.65	1.47±1.62	1.14±0.73	0.59±0.40	2.68±1.57
**MH Mean±SD**	0.78±0.47	0.93±0.63	1.18±0.84	1.10±0.82	0.93±0.53	0.71±0.40
**LH Mean±SD**	1.33±0.36	1.59±0.57	1.26±0.95	0.96±0.51	0.96±0.61	1.08±0.67
**TA Mean±SD**	1.05±0.74	0.98±0.62	0.94±0.59	3.02±1.66	1.15±0.98	1.34±1.00
**RF Mean±SD**	0.95±0.73	0.99±0.62	0.92±0.45	1.27±0.78	0.78±0.59	1.02±0.81
**VM Mean±SD**	0.94±0.39	1.42±0.51	1.47±1.04	0.65±0.39	0.62±0.21	0.93±0.72
**GM Mean±SD**	0.89±0.65	1.80±2.05	1.14±0.79	1.44±1.10	0.86±0.68	1.41±1.12
