# Supplementary material for: Altered muscle activation patterns (AMAP): an analytical tool to compare muscle activity patterns of hemiparetic gait with a normative profile
Source: J Neuroeng Rehabil. 2019 Jan 31;16:21. doi: 10.1186/s12984-019-0487-y (PMC6357420; doi:10.1186/s12984-019-0487-y)
Supplement: Supplementary file 1 — Figure S1. AMAP scores for all healthy individuals at all four walking speeds are provided. Figure S2. Average and SD of healthy individuals’ EMG patterns at all walking speeds. Table S1. EMG patterns for healthy individuals at all four walking speeds. Table S2. EMG patterns for stroke survivors at self-selected walking speeds. Table S3. Total AMAP scores for stroke survivors at their self-selected walking speeds (ZIP 1538 kb) [file 12984_2019_487_MOESM1_ESM.zip › Supplimentry Figure1_Final.pdf]

(A)

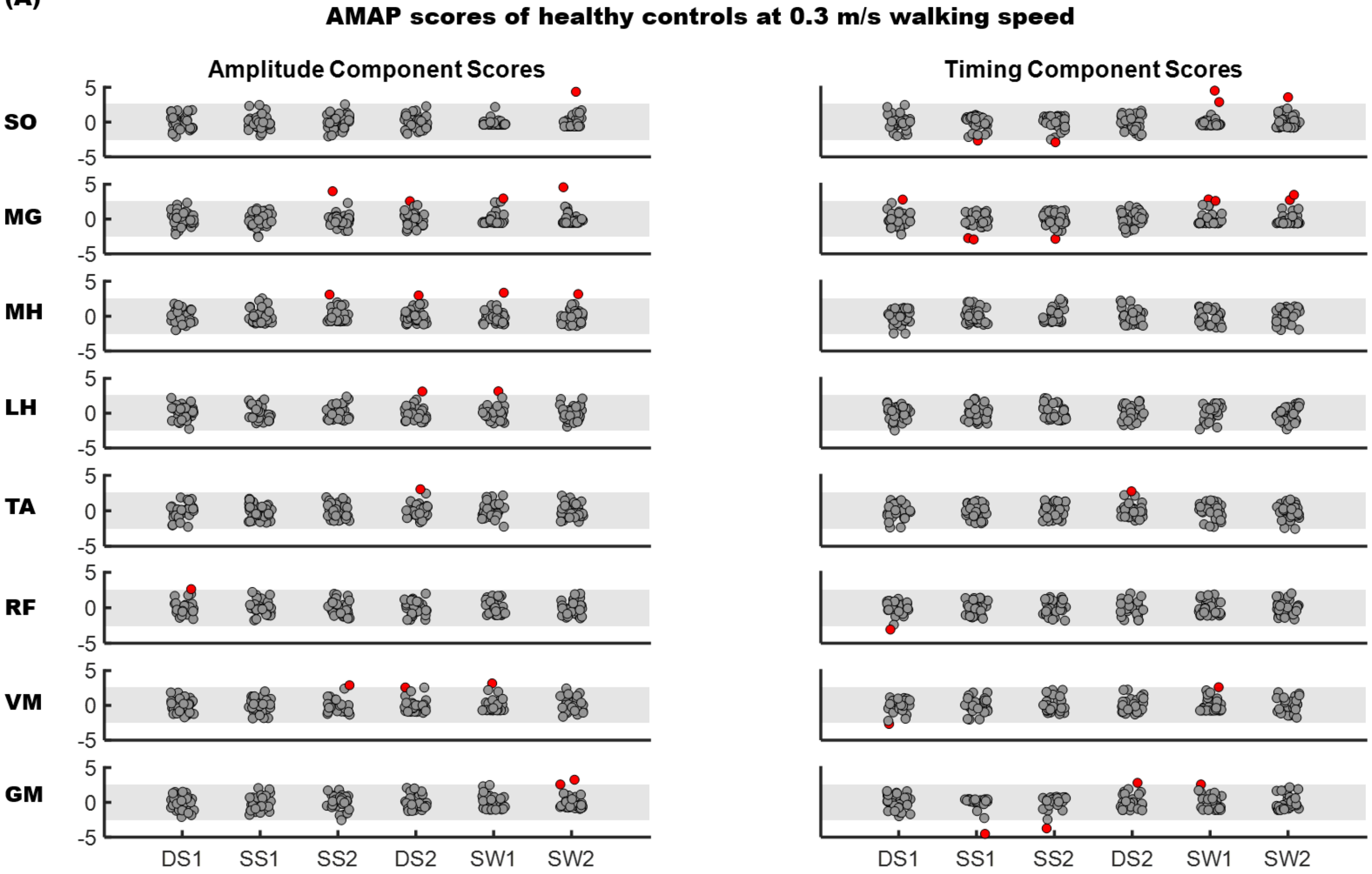
AMAP scores of healthy controls at 0.3 m/s walking speed
Amplitude Component Scores
Timing Component Scores
SO
MG
MH
LH
TA
RF
VM
GM
5
0
-5
DS1
SS1
SS2
DS2
SW1
SW2

(B)

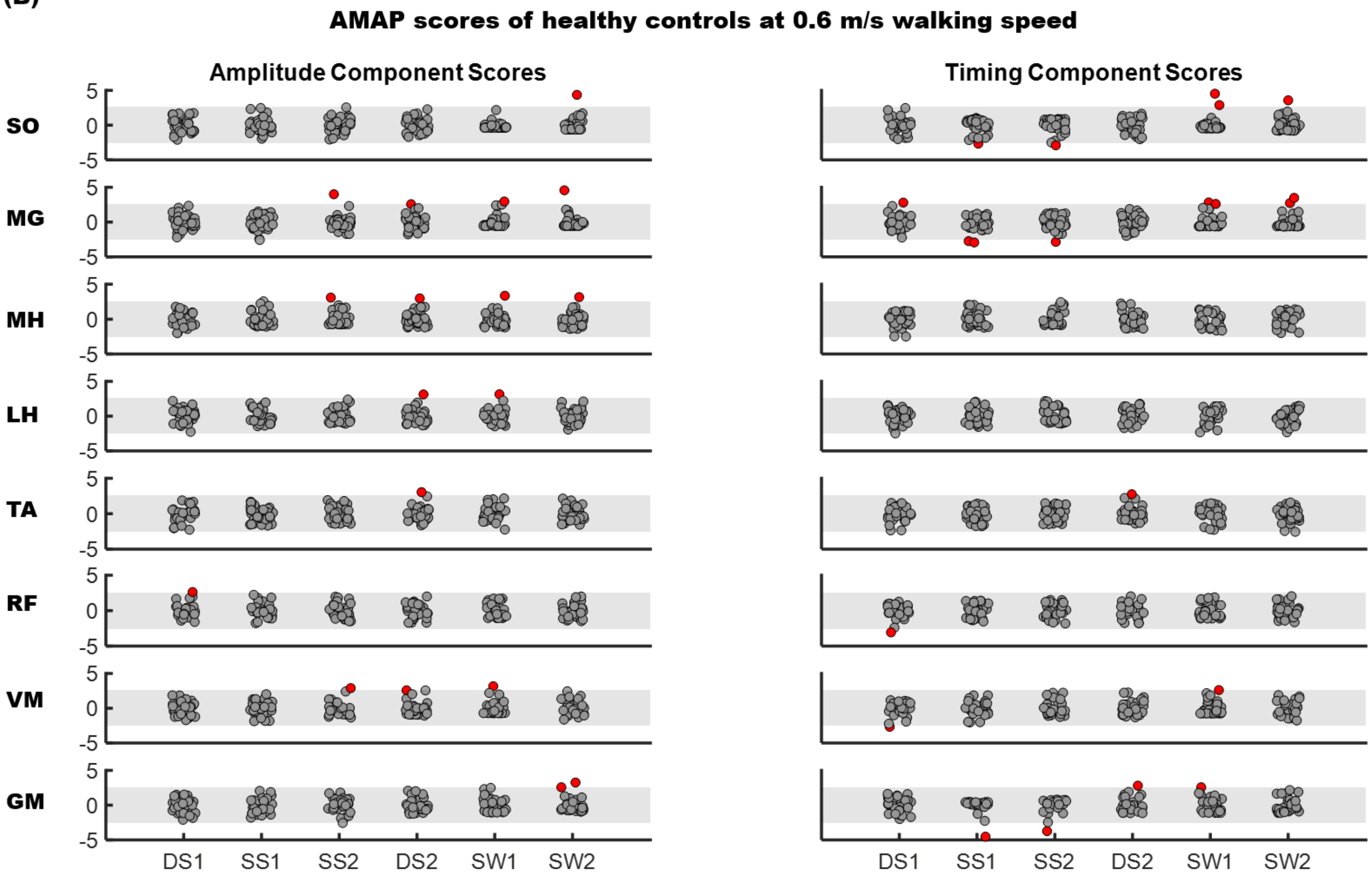

AMAP scores of healthy controls at 0.6 m/s walking speed
Amplitude Component Scores
Timing Component Scores
SO
MG
MH
LH
TA
RF
VM
GM
5
0
-5
DS1
SS1
SS2
DS2
SW1
SW2

(C)

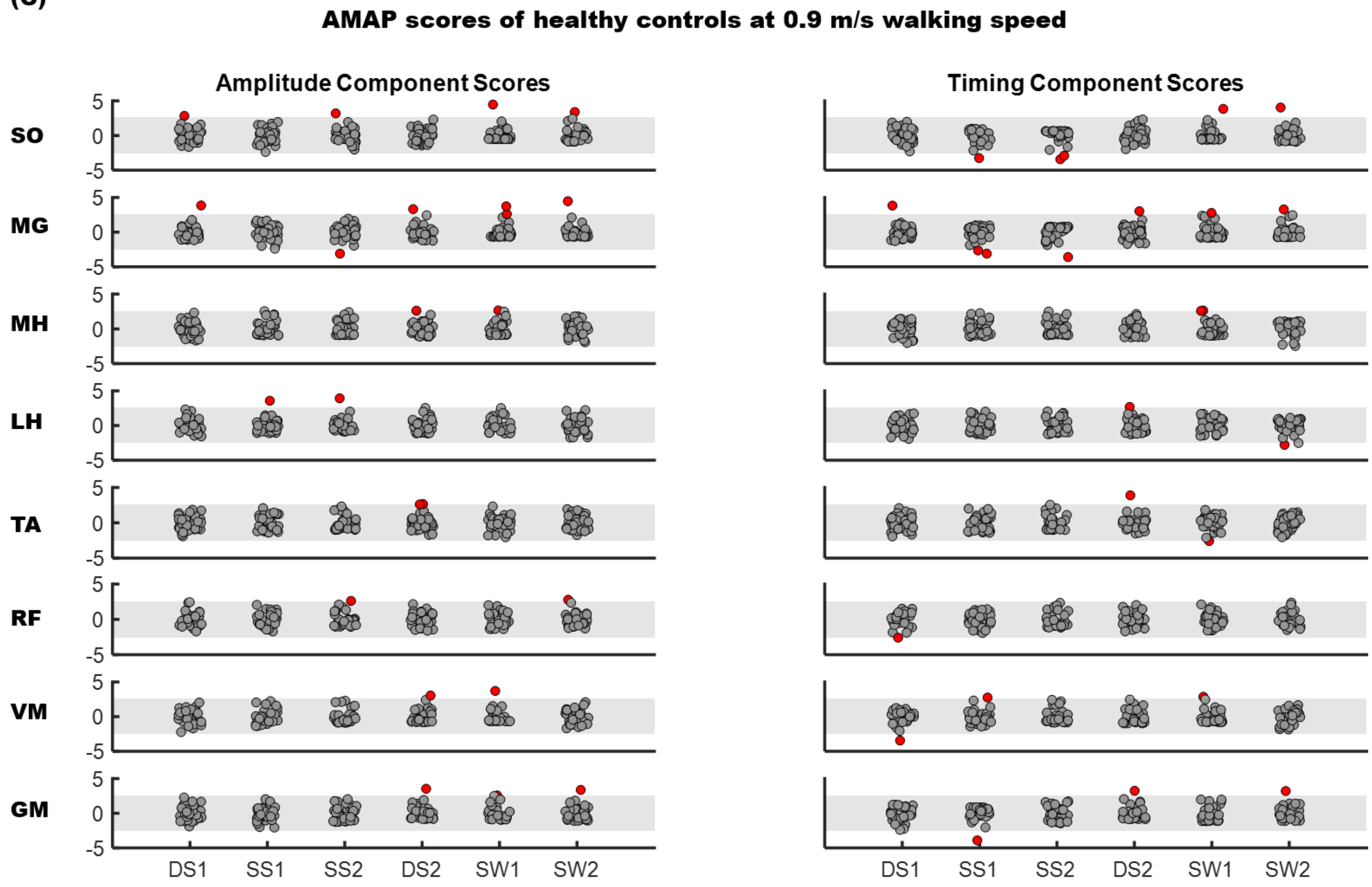

AMAP scores of healthy controls at 0.9 m/s walking speed
Amplitude Component Scores
Timing Component Scores
SO
MG
MH
LH
TA
RF
VM
GM
5
0
-5
DS1
SS1
SS2
DS2
SW1
SW2

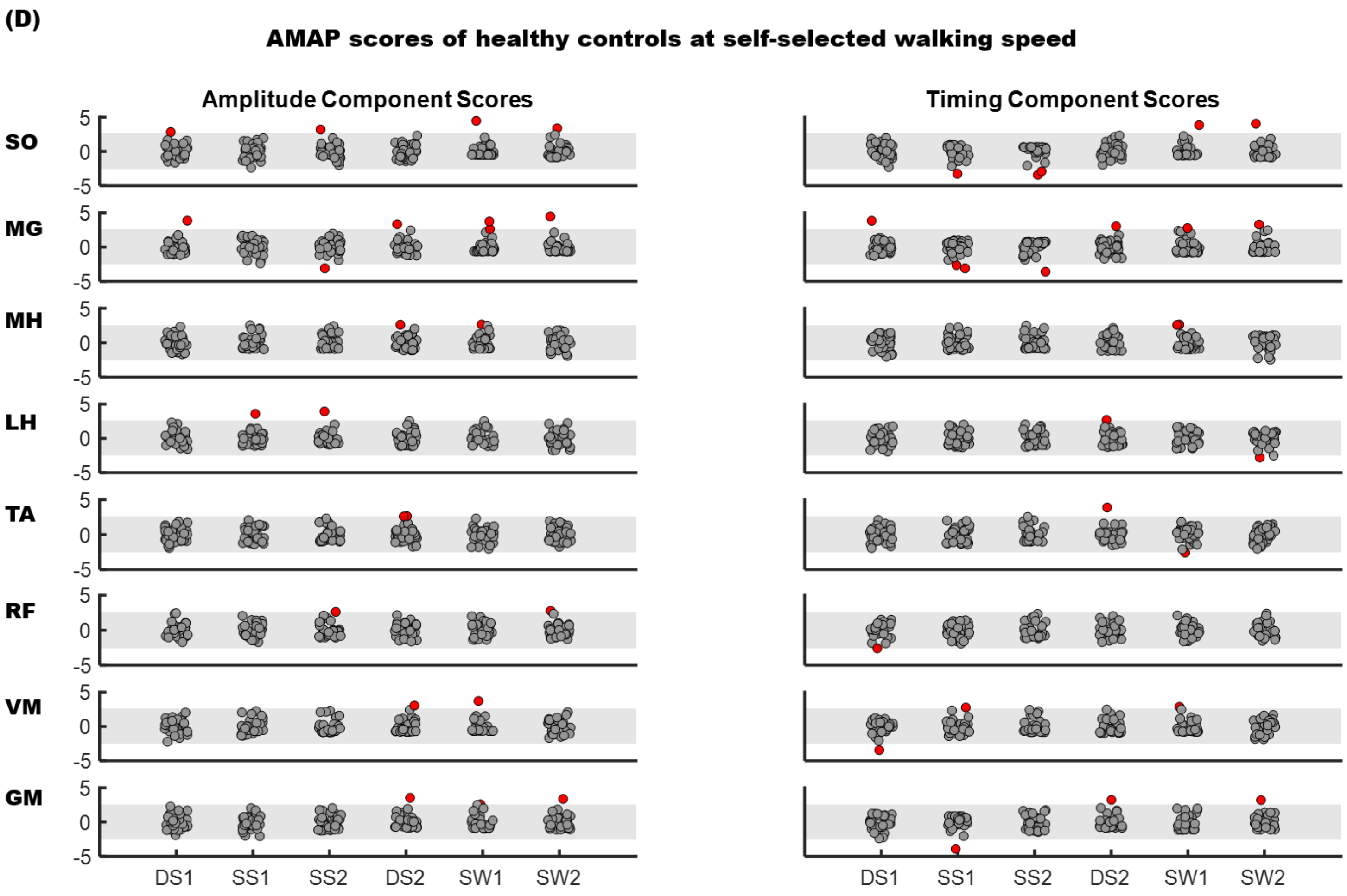

Figure S1. AMAP scores of healthy controls for magnitude and timing components of each muscle at (A) 0.3m/s, (B) 0.6m/s, (C) 0.9m/s, (D) self-selected walking speeds. The shaded gray area is the normal range of AMAP scores (±2.57). Each dot within a region of gait cycle represents score of a stroke survivor, and solid red dots represent the subjects with scores outside the “normal” window of ±2.57.
