# Supplementary material for: Altered muscle activation patterns (AMAP): an analytical tool to compare muscle activity patterns of hemiparetic gait with a normative profile
Source: J Neuroeng Rehabil. 2019 Jan 31;16:21. doi: 10.1186/s12984-019-0487-y (PMC6357420; doi:10.1186/s12984-019-0487-y)
Supplement: Supplementary file 1 — Figure S1. AMAP scores for all healthy individuals at all four walking speeds are provided. Figure S2. Average and SD of healthy individuals’ EMG patterns at all walking speeds. Table S1. EMG patterns for healthy individuals at all four walking speeds. Table S2. EMG patterns for stroke survivors at self-selected walking speeds. Table S3. Total AMAP scores for stroke survivors at their self-selected walking speeds (ZIP 1538 kb) [file 12984_2019_487_MOESM1_ESM.zip › Supplimentry Figure2_Final.pdf]

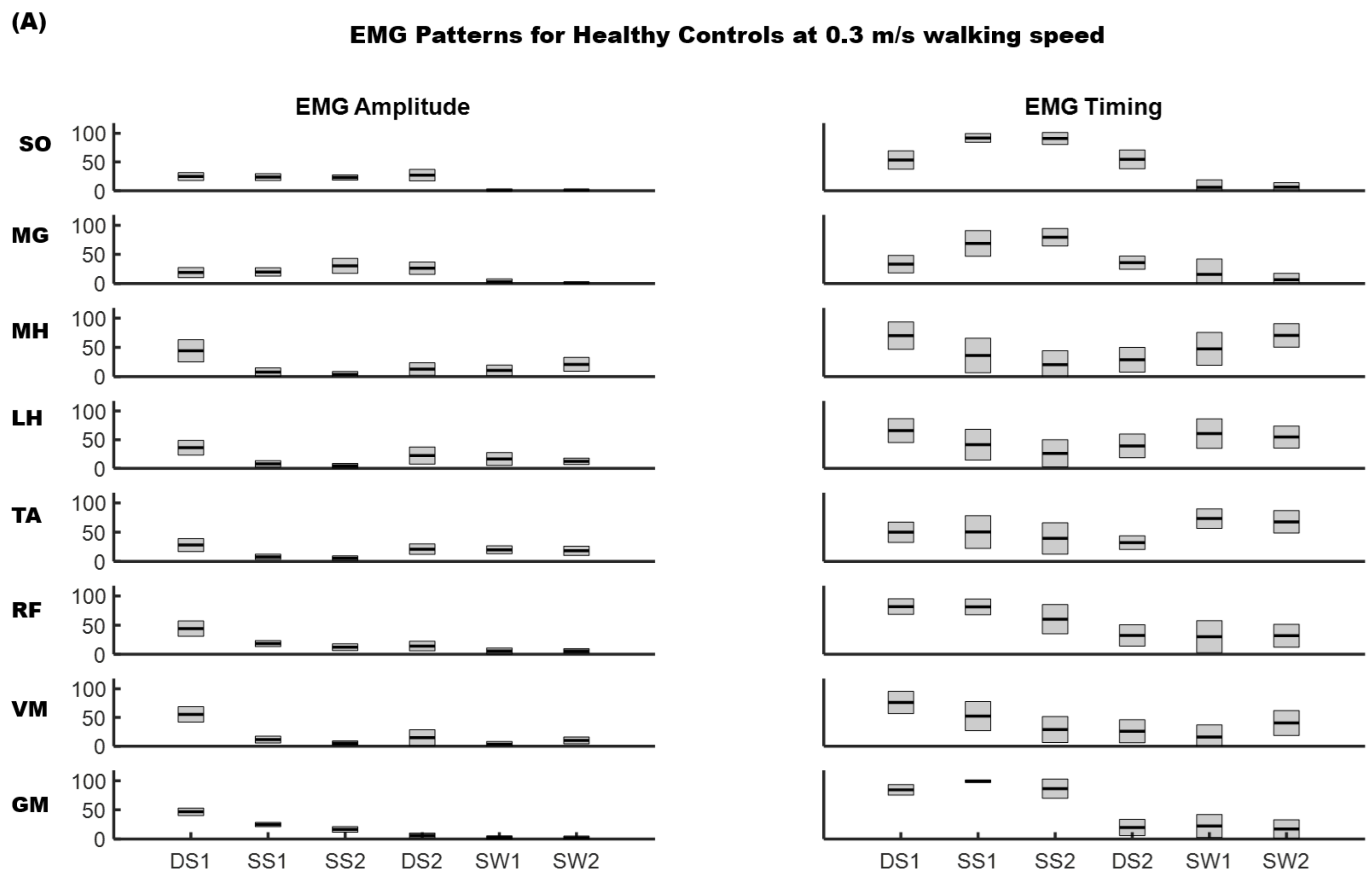
(A)
EMG Patterns for Healthy Controls at 0.3 m/s walking speed
EMG Amplitude
EMG Timing
SO
MG
MH
LH
TA
RF
VM
GM
100
50
0
DS1
SS1
SS2
DS2
SW1
SW2

(B)

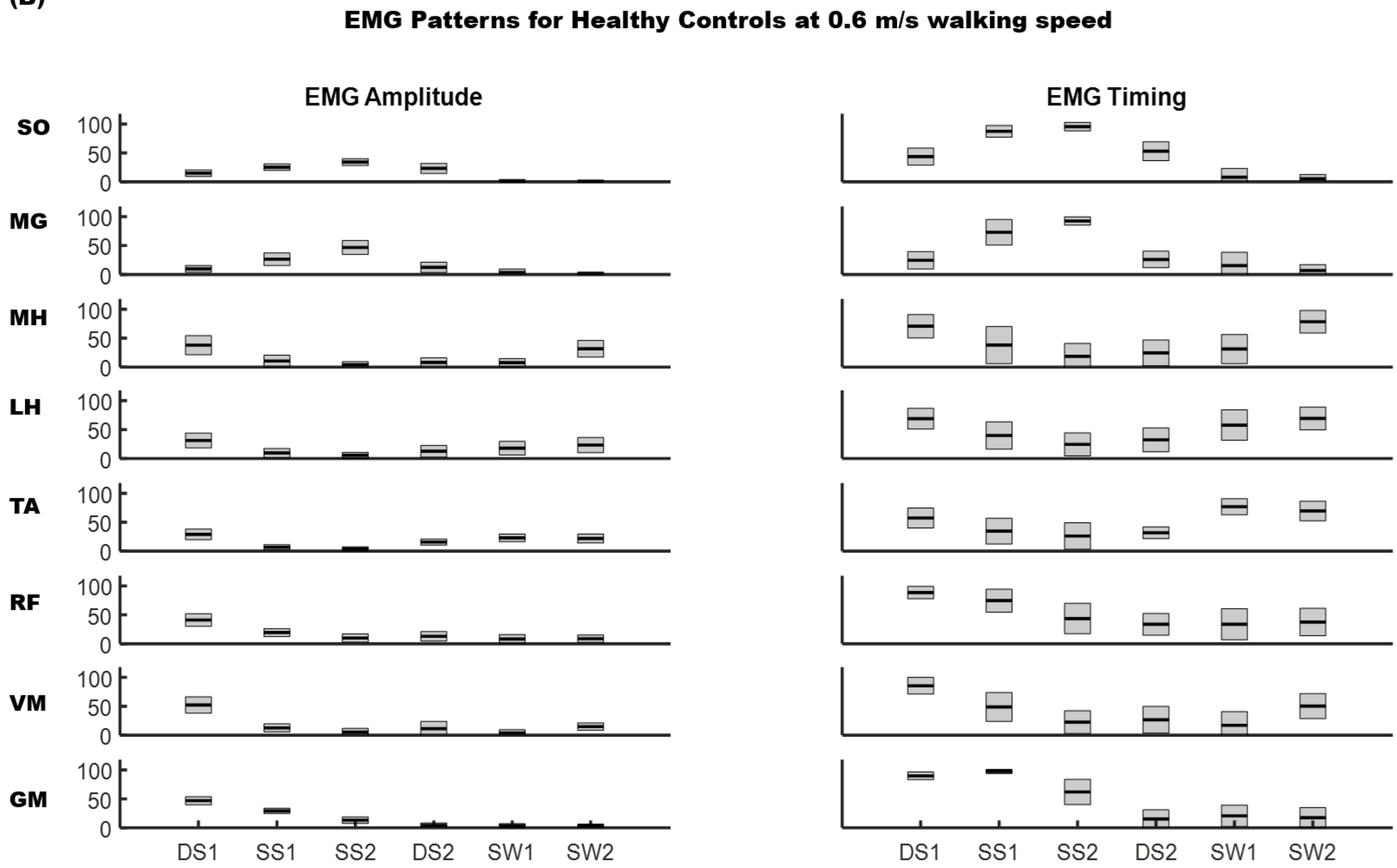
EMG Patterns for Healthy Controls at 0.6 m/s walking speed
EMG Amplitude
EMG Timing
SO
MG
MH
LH
TA
RF
VM
GM
100
50
0
DS1
SS1
SS2
DS2
SW1
SW2

(C)

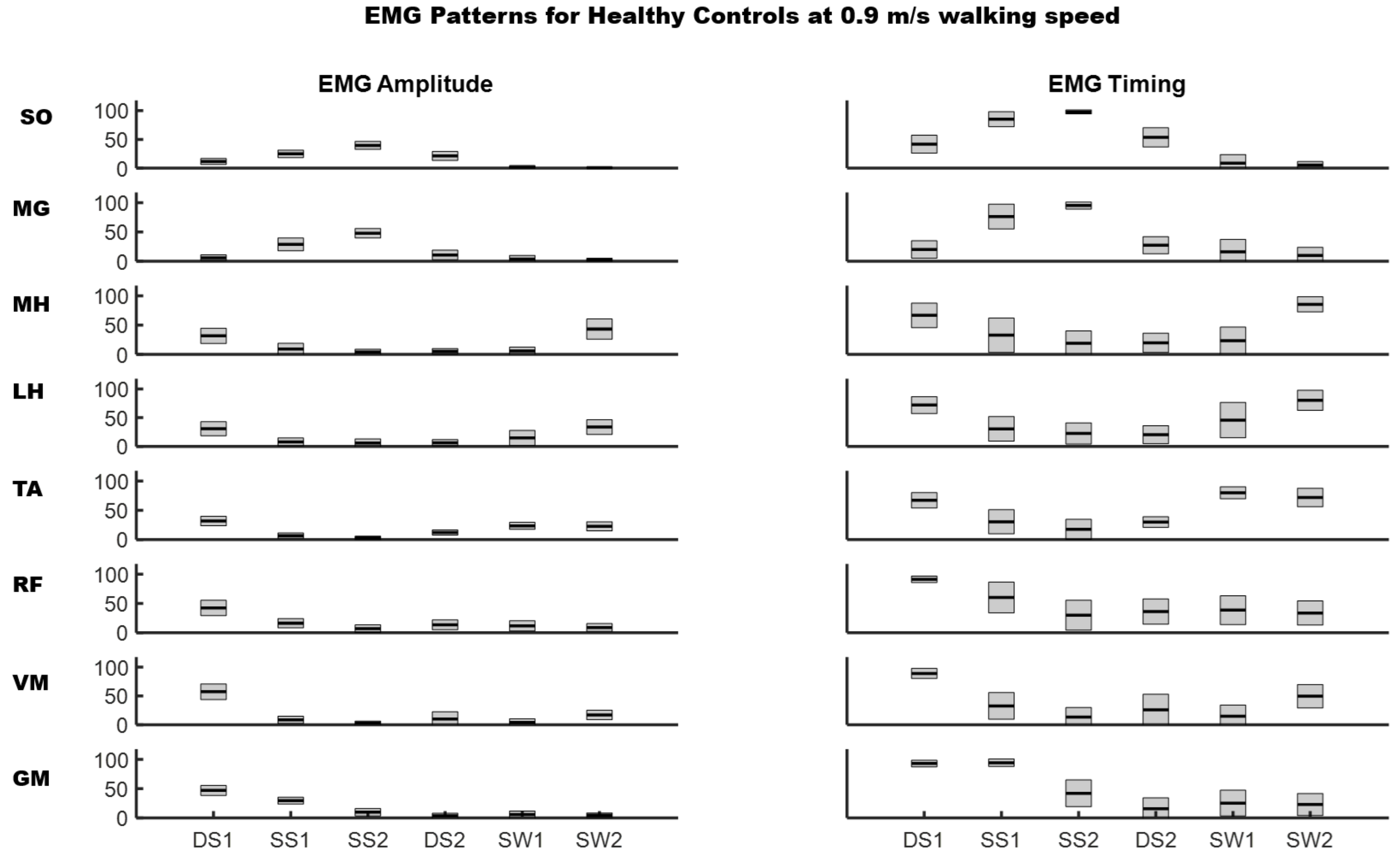
EMG Patterns for Healthy Controls at 0.9 m/s walking speed
EMG Amplitude
EMG Timing
SO
MG
MH
LH
TA
RF
VM
GM
100
50
0
DS1
SS1
SS2
DS2
SW1
SW2

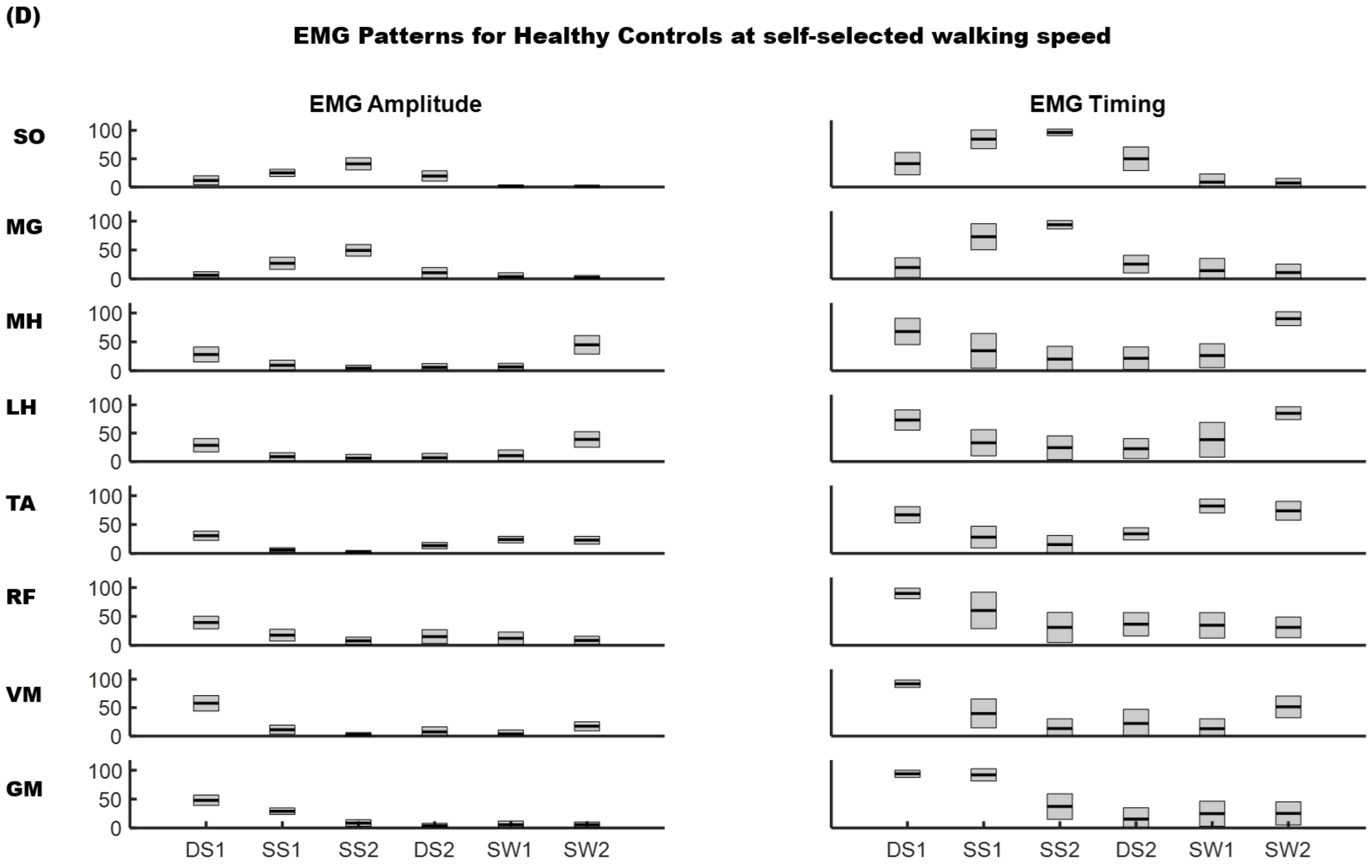

Figure S2. EMG patterns of healthy controls for amplitude and timing components of each muscle at (A) 0.3m/s, (B) 0.6m/s, (C) 0.9m/s, (D) self-selected walking speeds. The solid black line within each bar represents the mean for each region and the shaded gray bar is the ±SD. The regions with solid black line closer to zero and small ±SD represent typically “off” activity and regions with solid black line further away from zero

represent increase in EMG activity timing and amplitude. However, if the regions have large ±SD, this would represent the sub-phases with large inter-individual variability in healthy population.
